# Metastable Ta_2_N_3_ with highly tunable electrical conductivity *via* oxygen incorporation†

**DOI:** 10.1039/d1mh00017a

**Published:** 2021-04-01

**Authors:** Chang-Ming Jiang, Laura I. Wagner, Matthew K. Horton, Johanna Eichhorn, Tim Rieth, Viktoria F. Kunzelmann, Max Kraut, Yanbo Li, Kristin A. Persson, Ian D. Sharp

**Affiliations:** Walter Schottky Institute and Physics Department, Technische Universität München 85748 Garching Germany sharp@wsi.tum.de; Energy Technologies Area, Lawrence Berkeley National Laboratory Berkeley CA 94720 USA; Department of Materials Science and Engineering, University of California, Berkeley Berkeley CA 94720 USA; Institute of Fundamental and Frontier Sciences, University of Electronic Science and Technology of China Chengdu 610054 P. R. China

## Abstract

The binary Ta–N chemical system includes several compounds with notable prospects in microelectronics, solar energy harvesting, and catalysis. Among these, metallic TaN and semiconducting Ta_3_N_5_ have garnered significant interest, in part due to their synthetic accessibility. However, tantalum sesquinitride (Ta_2_N_3_) possesses an intermediate composition and largely unknown physical properties owing to its metastable nature. Herein, Ta_2_N_3_ is directly deposited by reactive magnetron sputtering and its optoelectronic properties are characterized. Combining these results with density functional theory provides insights into the critical role of oxygen in both synthesis and electronic structure. While the inclusion of oxygen in the process gas is critical to Ta_2_N_3_ formation, the resulting oxygen incorporation in structural vacancies drastically modifies the free electron concentration in the as-grown material, thus leading to a semiconducting character with a 1.9 eV bandgap. Reducing the oxygen impurity concentration *via* post-synthetic ammonia annealing increases the conductivity by seven orders of magnitude and yields the metallic characteristics of a degenerate semiconductor, consistent with theoretical predictions. Thus, this inverse oxygen doping approach – by which the carrier concentration is reduced by the oxygen impurity – offers a unique opportunity to tailor the optoelectronic properties of Ta_2_N_3_ for applications ranging from photochemical energy conversion to advanced photonics.

## Abstract 2

Metastable Ta_2_N_3_ with bixbyite structure is directly deposited by reactive magnetron sputtering. Concerted experimental and computational efforts reveal the crucial role of oxygen impurity in both the synthesis and in tuning the electronic structure.

## Graphical abstract

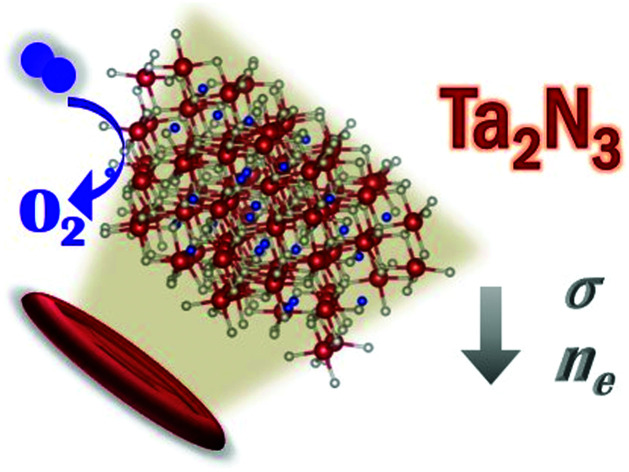

**New concepts**

Transition metal nitrides represent a versatile chemical space for creating new functional materials with tunable properties for a variety of applications. However, synthetic inaccessibility greatly hampers the technological development of functional nitrides – not only does the strong bonding of N_2_ molecules introduce kinetic barriers during synthesis, but it also makes many nitride compounds metastable against decomposition. In this work, we leverage the stronger electronegativity of oxygen to inductively stabilize higher metal oxidation states, such that the metallic mononitrides are converted to nitrogen-rich and semiconducting polymorphs. With this concept, the metastable and rarely reported Ta_2_N_3_ can be directly prepared by reactive magnetron sputter deposition, with the intentional addition of O_2_ into the sputtering environment. On the other hand, the extent of oxygen incorporation can be controlled and used to tailor the optoelectronic properties of the parent nitride materials. Such dual role of oxygen, in both facilitating the formation of metastable nitrides and tuning their functional characteristics, opens avenues of developing the previously underexplored nitride chemical space.

## Introduction

Metal nitrides offer intriguing prospects for a variety of applications, including solid-state lighting,^1^ integrated circuits,^2^ thermoelectrics,^3^ superconductors,^4^ and photocatalysis.^5–7^ The moderate electronegativity of nitrogen (*χ*_N_ = 3.04), compared to oxygen (*χ*_O_ = 3.44), results in a mixed ionic/covalent bonding character in these compounds. For such nitrides, the strong electrostatic interaction between N^3−^ and metal cations translates to higher lattice cohesive energies, which manifest in their mechanical hardness and refractoriness.^8^ On the other hand, the N 2p energy levels are located in closer proximity to the metal electronic states, thus giving rise to a higher degree of orbital hybridization and improved charge transport properties compared to the corresponding metal oxides. While metal oxides are typically dielectrics or semiconductors, the electronic structure of transition metal nitrides is strongly affected by the nitrogen content and spans from metallic to semiconducting. Mononitrides of early transition metal elements, such as TiN, ZrN and TaN, have been widely utilized as wear-resistant coatings and metal diffusion barriers in microelectronics, and their excellent electrical conductivities can be attributed to the partially occupied metal d states.^9^ In comparison, the nitrogen-rich compounds (*e.g.* Ti_3_N_4_, Zr_3_N_4_, and Ta_3_N_5_) that adopt the highest possible metal oxidation states are expected to behave as semiconductors due to the energy gap separating the N 2p states from the unoccupied metal d states.^10^ Therefore, transition metal nitrides represent a versatile and promising chemical space, from which new functional materials can be developed.

Hundreds of metal nitride phases are predicted to be (meta)stable by density functional theory (DFT) computation and await experimental realization.^11,12^ The synthesis of N-rich metal nitrides, however, proves to be a challenging task for two reasons. First, formation of N-rich nitrides requires high metal oxidation states, which are not as readily achievable as in the corresponding oxides due to the electronegativity difference between nitrogen and oxygen. Accordingly, a common synthesis strategy is to form the corresponding oxide prior to the nitridation step, as in the case of converting Ta_2_O_5_ to Ta_3_N_5_ by ammonolysis.^13^ Second, N-rich nitrides generally have low formation enthalpies that originate from the strong bonding of N_2_ molecules (964 kJ mol^−1^).^14^ To overcome this, many chemical vapor deposition (CVD) techniques employ more reactive nitrogen precursors such as azide, urea, or ammonia for preparing nitride compounds. Alternatively, the low chemical reactivity of N_2_ can be overcome through high pressure and high temperature (HPHT) synthesis routes, which have successfully yielded selected N-rich metal nitrides, including Ti_3_N_4_ and Zr_3_N_4_,^15,16^ though this technique lacks industrial scalability and is largely incompatible with thin film synthesis. To navigate the underexplored nitride chemical space effectively, there is a pressing need to identify key experimental parameters for synthesizing N-rich transition metal nitrides at low process temperatures and pressures.

The binary Ta–N system has a rich phase diagram with a wide range of N/Ta stoichiometries and several phases possessing properties of significant technological relevance.^17–19^ Among these, the orthorhombic Ta_3_N_5_ represents the most N-rich phase with the highest possible +5 formal oxidation state of Ta. This n-type semiconductor features a 2.1 eV direct bandgap and suitable band edge positions for driving water splitting reactions. Thus, it has garnered extensive research interest for solar energy conversion.^6,20–22^ The most common synthesis method of Ta_3_N_5_ is through nitridation of Ta_2_O_5_ in an ammonia flow at 850–1000 °C, though recently there are reports of direct Ta_3_N_5_ thin film deposition *via* atomic layer deposition^23^ and reactive magnetron sputtering.^24^ In contrast, tantalum mononitride (TaN) can form different polymorphs, including the δ-, ε-, and θ-phases, which exhibit high electrical conductivities. Thin films of these materials are often prepared by reactive sputtering and are widely used as diffusion barriers between copper and silicon within integrated circuits. The possibility to form non-stoichiometric TaN by adding Ta or N vacancies should be noted, in that the composition range of TaN_*x*_ can vary from *x* = 0.8 to 1.4.^25^ This is important since increasing the nitrogen content diminishes the Ta 5d orbital overlap and consequently reduces the electrical conductivity. As an even more metal-rich phase, β-Ta_2_N, can be obtained by sintering Ta in N_2_ or NH_3_. The structure of this compound can be depicted as an hcp tantalum lattice with nitrogen occupying half of the octahedral interstitial sites.^26^

Despite the scientific and technological advancements of these tantalum nitride compounds, tantalum sesquinitride (Ta_2_N_3_) remains relatively unexplored. The first report of Ta_2_N_3_ dates back to 1968 by Coyne *et al.*^27^ However, in the ensuing 50 years there have only been a handful of reports related to the cubic bixbyite phase of Ta_2_N_3_. For example, in 2016, Salamon *et al.* reported bixbyite Ta_2_N_3_ grown by reactive sputtering.^28^ They found that transport characteristics were dominated by a combination of nitrogen excess in as-grown material and grain boundary barriers that were modified as a function of the post-synthetic vacuum annealing temperature. In contrast to the report of Coyne, their experimental and computational results indicated that Ta_2_N_3_ exhibits metallic conduction. However, neither the role of incorporated oxygen within the Ta_2_N_3_ phase on electronic structure and transport, nor its critical impact on metastability were addressed in that work. In addition to bixbyite-type Ta_2_N_3_, orthorhombic η-Ta_2_N_3_ has been synthesized using the HPHT method.^29^ First principles calculations also predicted another tetragonal polymorph of Ta_2_N_3_ that undergoes phase transformation to η-Ta_2_N_3_ at 7.7 GPa.^30^ However, the present work will focus on the cubic bixbyite phase of Ta_2_N_3_, which can be deposited in thin film form. Given the technological importance of the Ta–N system for diverse applications, scalable synthesis and detailed characterization of this lesser-known cubic bixbyite Ta_2_N_3_ phase provides interesting prospects for creating tunable materials with intermediate properties for new applications, as well as for understanding the key factors that allow synthesis of such metastable nitride phases.

Herein, we report the non-equilibrium deposition of bixbyite-type Ta_2_N_3_ thin films by reactive magnetron sputtering and characterize their optoelectronic properties. We find that the formation of metastable Ta_2_N_3_ hinges upon the presence of a small amount (0.65%) of O_2_ in the process gas mixture, which provides an inductive effect to stabilize higher oxidation states of Ta and suppress formation of metallic TaN. The presence of O_2_ during synthesis leads to the incorporation of 11.6 at% oxygen in the as-grown films. However, we find that reactive annealing in NH_3_ can be applied to reduce the concentration of oxygen impurities. Optical and electrical measurements, supported by density functional theory, reveal that stoichiometric Ta_2_N_3_ is a degenerate semiconductor with a ∼1.9 eV internal bandgap. However, the effective electron density in the conduction band is greatly reduced by the incorporation of oxygen at structural vacancy sites. Using this inverse oxygen doping approach to compensate free carriers of the intrinsically metallic film, we demonstrate that a conductivity variation of seven orders of magnitude can be readily achieved *via* post-synthetic reactive annealing. Not only do these findings resolve previously conflicting reports regarding the electronic structure of Ta_2_N_3_, but they also allow the tuning of the electrical characteristics from metallic to semiconducting character by varying the oxygen content. Thus, Ta_2_N_3_ with controlled oxygen content represents a versatile system with tunable metallic and semiconducting character for potential applications ranging from advanced plasmonics to photoelectrochemical energy conversion.

## Results and discussion

**Fig. 1:**
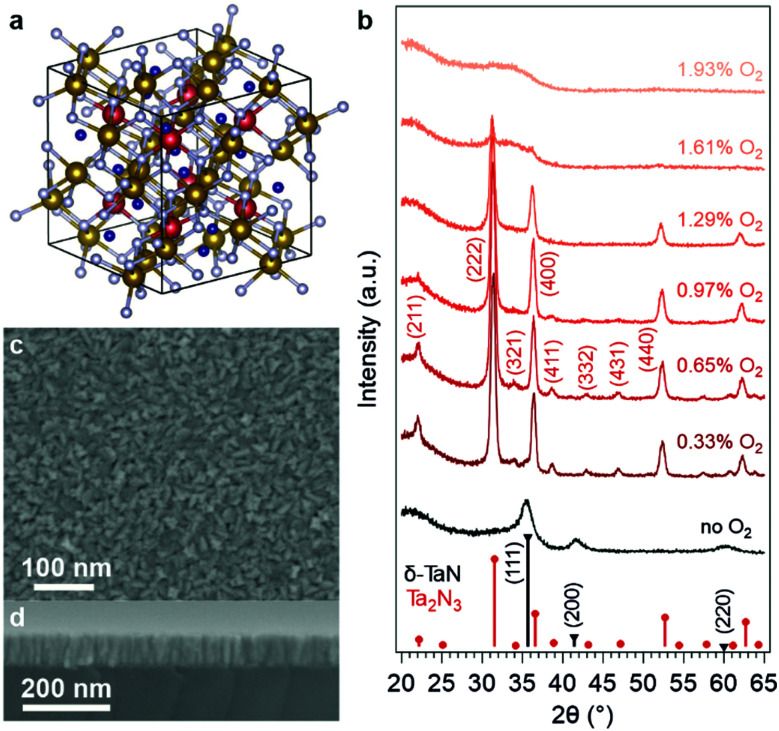
(a) Crystal model of bixbyite-type Ta_2_N_3_. The two non-equivalent Ta sites (Wyckoff 8b and 24d) are respectively labeled in red and brown, whereas nitrogen atoms are labeled in light gray. The structural nitrogen vacancy sites (Wyckoff 16c) are labelled as blue dots. The thin lines indicate the cubic unit cell of bixbyite with lattice constant *a*_0_ = 9.8205 Å. (b) Grazing-incidence XRD patterns of as-grown Ta_2_N_3_ thin films deposited by reactive magnetron sputtering with various amounts of O_2_ in the process gas mixture. Gray and red sticks: Bragg-reflection positions and intensities of δ-TaN and Ta_2_N_3_, respectively. (c) Plan view and (d) cross-sectional view SEM images of an as-grown Ta_2_N_3_(O) thin film. The sample was deposited on silicon at 500 °C growth temperature with 0.65% O_2_ in the process gas mixture.

Fig. 1a illustrates the bixbyite structure (space group *Ia*3̄) of Ta_2_N_3_, which can be considered as an ordered-defect variant of the CaF_2_-type. While in the fluorite structure every cation is equivalent and coordinated with 8 anions, in Ta_2_N_3_ there exist two types of six-fold N-coordinated Ta cations, which then stack to form an fcc array. Accordingly, the CaF_2_ unit cell is expanded to a 2 × 2 × 2 superstructure so that the ordered arrangement of unoccupied voids in the bixbyite structure is accounted for. For each Ta at the 8b Wyckoff site, there are two nitrogen atoms absent from the 16c sites along a body diagonal; for each Ta at the 24d site, the two structural nitrogen vacancies are located along a face diagonal. In the first report of Ta_2_N_3_, the authors indexed the X-ray diffraction pattern to an fcc unit cell with lattice constant *a*_0_ ∼ 5.011 Å.^27^ Electron diffraction identified additional peaks corresponding to lattice plane spacings of 4.08, 2.13, and 1.95 Å, which were originally assigned to impurity phases. Upon reexamination, it is clear that these diffraction peaks stem from the (211), (332), and (431) lattice planes of the bixbyite Ta_2_N_3_ (Fig. 1b)—the lowering of lattice symmetry gives rise to these weak peaks observed not only in the Ta_2_N_3_ diffraction pattern, but also in C-Y_2_O_3_, In_2_O_3_, and U_2_N_3_ that all adopt the same bixbyite structure.^31–33^ In 2004, Ganin *et al.* prepared a microcrystalline Ta_2_N_3_ thin film by plasma-enhanced CVD, and indexed it to the bixbyite structure for the first time with a lattice constant of 9.8205 Å.^17^

In this work, thin films of tantalum nitride were deposited on Si(100) and amorphous SiO_2_ substrates by reactive magnetron sputtering. This non-equilibrium deposition technique generates highly reactive atomic nitrogen by cracking N_2_ molecules with plasma, thus enabling metastable nitride phases to be synthetically accessed.^34,35^ The process gas comprised Ar and N_2_ (flow rate ratio 1 : 2) with a ∼6.7 mTorr total pressure. Importantly, a controlled small dose of oxygen in the process gas mixture was found to be imperative to the formation of bixbyite Ta_2_N_3_. To investigate this, a series of thin films was grown at 500 °C with 0–1.9% O_2_ content in the process gas (Fig. 1b). In the absence of intentionally added oxygen, the appearance of the film was optically dense and metallic (Fig. S1 in ESI†), with X-ray diffraction (XRD) indicating formation of the δ-TaN phase. The film changed to a brown-orange color with merely 0.33% O_2_ present in the process gas. This optical change was accompanied by the evolution of the XRD pattern towards that of bixbyite Ta_2_N_3_. The highest crystallinity was obtained at 0.65% O_2_ concentration, which also yielded no residual δ-TaN phase. However, at higher oxygen flow rates, the weaker diffraction peaks that are unique for the low symmetry defect-ordered bixbyite structure were reduced and disappeared completely with 1.29% O_2_ in the process gas. At even higher oxygen concentrations, the films became amorphous, likely due to the formation of a disordered oxynitride. As discussed below, this finding is consistent with computational predictions of the above-hull energy of the stoichiometric Ta_2_N_3_O phase, which exceeds the tolerance of the system to form a metastable crystalline phase and instead favors a disordered amorphous phase.

For the as-grown sample deposited with 0.65% O_2_ in the process gas mixture, SEM images revealed closely packed crystallites of ∼10 nm size that formed a homogeneous thin film covering the substrate (Fig. 1c). Cross-sectional imaging indicated that individual crystallites extend across the entire thickness of the film, suggesting a columnar growth mode (Fig. 1d). The compactness of the film was verified by X-ray reflectivity (XRR, Fig. S3, ESI†), which yielded a 10.64 ± 0.18 g cm^−3^ mass density that is only 6% smaller than the 11.33 g cm^−3^ bulk density of bixbyite Ta_2_N_3_. The film thickness, also determined *via* XRR, was 91.2 ± 0.3 nm for the 105 min growth time. In addition to SiO_2_ and Si substrates, the versatility of reactive magnetron sputtering also enabled the deposition of as-grown Ta_2_N_3_ thin films on GaN, Nb, and Ta substrates (Fig. S4, ESI†).

**Fig. 2:**
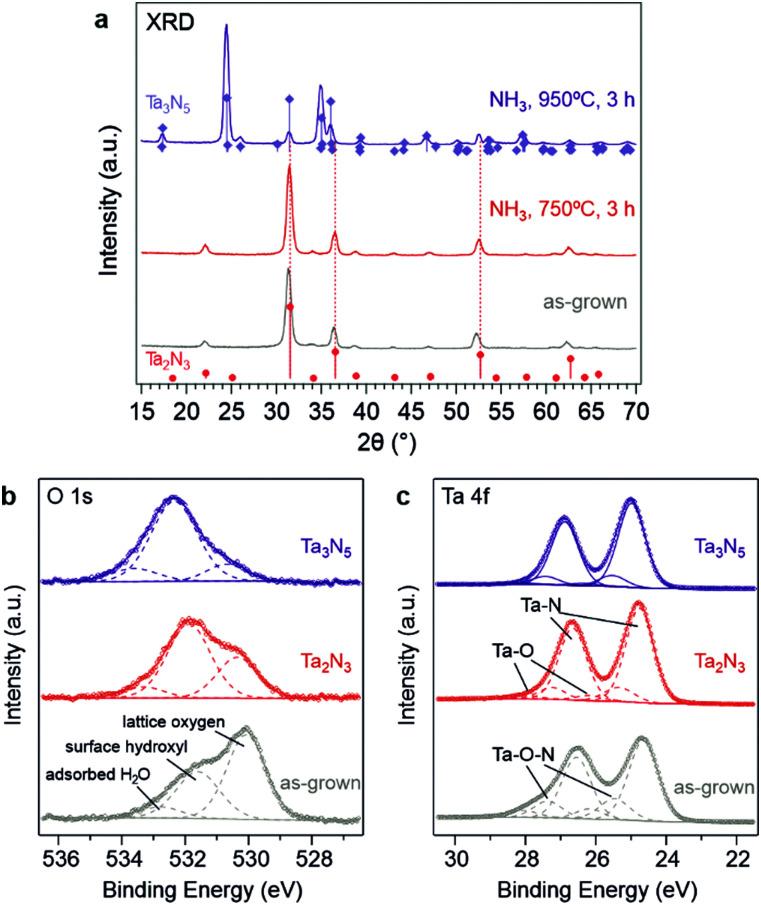
(a) XRD patterns of as-grown Ta_2_N_3_(O) on Si substrate and after NH_3_ annealing treatment at different temperatures. The blue and red sticks represent the reference pattern of orthorhombic Ta_3_N_5_ (PDF# 79-1533) and bixbyite-type Ta_2_N_3_, respectively. (b) O 1s and (c) Ta 4f region XPS spectra of the as-grown Ta_2_N_3_(O) and the Ta_2_N_3_ after NH_3_ annealing treatments.

The X-ray diffractogram measured from the as-grown films resembled the reference bixbyite Ta_2_N_3_ pattern reported by Ganin *et al.*,^17^ albeit with smaller diffraction angles (Fig. 2a and Fig. S4, ESI†). Because of the presence of O_2_ during the reactive sputtering process, a certain quantity of oxygen is incorporated into the films, which consequently increases the unit cell volume and reduces the diffraction angles. Given that the O 2p states are located at energetically lower energies than the N 2p states, an increasing oxygen content in the film should push the valence band maximum downward and thus increase the bandgap. Indeed, the optical absorption onset moved monotonically toward shorter wavelengths with increasing O_2_ concentration in the process gas (Fig. S2, ESI†). To elucidate the role of oxygen incorporation on the optoelectronic properties of as-grown Ta_2_N_3_, quantitative elemental analysis was performed by combining Rutherford backscattering spectroscopy (RBS) and elastic recoil detection (ERD). The Ta, N, and O contents in the as-grown film deposited with 0.65% O_2_ in the process gas were found to be 33.3, 51.7, and 11.6 atomic%, respectively (Table S1, ESI†). Given that the 1.56 N/Ta ratio is already greater than the expected 1.50 value for stoichiometric Ta_2_N_3_, the oxygen in as-grown Ta_2_N_3_ is more likely to occupy interstitial sites rather than substituting nitrogen. Indeed, in the bixbyite structure, the structural nitrogen vacancies at the 16c Wyckoff sites are suitable for accommodating extra oxygen atoms, which would yield a Ta_2_N_3_O stoichiometry for the case of full oxygen occupancy. Starting with the unit cell parameters of bixbyite-type Ta_2_N_3_ that are published by Ganin *et al.*,^17^ we added an oxygen to the 16c site and then employed DFT to calculate the relaxed structure with the PBEsol^36^ energy functional (Table S2, ESI†). Not only does the resulting unit cell maintain a bixbyite-like diffraction pattern (Fig. S8, ESI†), the lattice constant also increases slightly to 9.9453 Å, consistent with the experimental observation. Therefore, the as-grown tantalum nitride film is hereafter referred as Ta_2_N_3_(O), indicating that the crystal structure resembles the bixbyite-type Ta_2_N_3_ but with oxygen predominantly occupying the structural nitrogen vacancies to varying degrees. To the best of our knowledge, such an ordered oxygen impurity incorporated variant of Ta_2_N_3_ has never before been reported.

The chemical nature of O, Ta, and N in the as-grown Ta_2_N_3_(O) film was investigated by X-ray photoelectron spectroscopy (XPS). The main O 1s peak at a binding energy of ∼530.1 eV (Fig. 2b) has been previously assigned to lattice oxygen in Ta_3_N_5_, while the peaks at 531.6 eV and 532.8 eV are consistent with surface hydroxyl species and adsorbed water, respectively.^37^ The Ta 4f region exhibited a 1.9 eV spin–orbit splitting and can be deconvoluted to three spin–orbit split doublets. The first doublet at 24.7 and 26.6 eV represents the Ta_2_N_3_ phase, and the second doublet at 25.5 and 27.4 eV corresponds to a more highly oxidized state of tantalum arising from oxygen incorporation in the form of Ta_2_N_3_(O) oxynitride. The third doublet, with the highest binding energies at 26.2 and 29.1 eV, is consistent with the presence of a native tantalum oxide on the surface.

As described above, metallic δ-TaN is obtained if no O_2_ flow is used during the deposition, while amorphous oxynitride films are formed if the O_2_ flow is too high. Based on these results, we conclude that a small amount of oxygen was required to favor the formation of bixbyite-type Ta_2_N_3_ rather than δ-TaN during the reactive sputtering process. Interestingly, Rudolph *et al.* reported a similar phenomenon in the reactive sputter deposition of orthorhombic Ta_3_N_5_.^24^ In their work, the highest phase purity and crystallinity was achieved with 4.4 at% oxygen incorporated in the film through intentional addition of O_2_ in the process gas mixture. Both that work and the work presented here aim to synthesize N-rich tantalum nitride thin films by reactive sputtering in Ar/N_2_ mixtures, but the underlying reasons for the formation of two different phases (Ta_3_N_5_ and Ta_2_N_3_) are not immediately clear. The contrast may be attributed to the nucleation kinetics on the substrate, in that the initial seeding layer dictates the crystal phase in the following growth period. Nevertheless, in both cases the ability of a small amount of oxygen to transform δ-TaN into N-rich nitride phases is more than a coincidence. Analogous to the oxygen inductive effect in organic chemistry, it is hypothesized that lattice oxygen stabilizes Ta cations with formal oxidation states higher than the +3 state that is found in δ-TaN; for the cases of Ta_3_N_5_ and Ta_2_N_3_, average oxidation states of +5 and +4.5, respectively, must be chemically accessed.

We note that when sintering Ta_2_O_5_ in an NH_3_ atmosphere, completely eliminating oxygen anions has proven to be challenging. For example, Henderson *et al.* reported that the highest crystallinity of Ta_3_N_5_ obtained through ammonolysis contains 2.9 at% oxygen.^38^ The effects of oxygen substituent defects in Ta_3_N_5_ on electronic structure and photoluminescence have also attracted broad research interest.^39^ Rather than attempting to achieve pure nitride synthesis, we suggest that nitride-related research should leverage the impact of oxygen impurities in phase formation and stabilization, such that the transition metal nitride chemical space can be more effectively explored. However, as will be shown below, even a small amount of oxygen in the background can have a significant influence on the functional characteristics of tantalum nitrides. Given this dual role of oxygen, both in the synthesis of metastable nitrides and on their resulting electronic structure, establishing composition–structure–property relationships with a focus on the role of oxygen impurities within the parent material is key to the development of functional materials within this class. We note that the other Ta_2_N_3_ polymorph – the orthorhombic η-Ta_2_N_3_ obtained through the HPHT method – also contains oxygen impurities with an O/(N + O) ratio of ∼0.05.^29^ Interestingly, a related theoretical study indicated that oxygen substitution into nitrogen sites could enhance the structural stability of this metastable compound.^40^

With the critical role of oxygen in mind, a strategy for reducing incorporated oxygen from the as-grown Ta_2_N_3_(O) films was essential for allowing characterization of the optoelectronic properties of stoichiometric Ta_2_N_3_, as well as for isolating the impact of oxygen on these properties. To this end, reactive annealing in flowing NH_3_ at 750 °C for 3 h was performed and proved to be effective. After such a treatment, the X-ray diffraction peaks shifted to higher angles that are consistent with the previously reported 9.8205 Å lattice constant of bixbyite Ta_2_N_3_ (Fig. 2a). Moreover, XPS results indicated that both the 530.1 eV O 1s peak and the tantalum oxynitride doublet in the Ta 4f region were greatly diminished (Fig. 2b and c). Elemental analysis by RBS and ERD revealed the Ta, N, and O contents to be 28.5, 63.5, and 5.8 at% after NH_3_ annealing treatment at 750 °C (Fig. S7 and Table S1, ESI†). Even though the incorporated oxygen was not entirely eliminated, its content was reduced to the range commonly found in, for example, intensively studied Ta_3_N_5_ films. Therefore, the sample after ammonolysis at 750 °C is hereafter referred to as Ta_2_N_3_ in order to distinguish it from the as-grown Ta_2_N_3_(O). At annealing temperatures higher than 750 °C, phase transformation from bixbyite-type Ta_2_N_3_ to orthorhombic Ta_3_N_5_ was observed: a mixed-phase XRD pattern was obtained after annealing in flowing NH_3_ at 845 °C for 3 h (Fig. S9, ESI†), and at 950 °C the conversion to phase pure Ta_3_N_5_ was complete. In the latter case, the core level binding energies of the Ta 4f_7/2_ and 4f_5/2_ peaks related to Ta–N bonding were ∼0.25 eV higher than their counterparts in bixbyite Ta_2_N_3_ (Fig. 2c). This can be attributed to the higher average Ta oxidation state in Ta_3_N_5_ (+5) compared to Ta_2_N_3_ (+4.5). It is worth pointing out that even after ammonolysis at 950 °C, the orthorhombic Ta_3_N_5_ still contained residual oxygen, as demonstrated by the O 1s and Ta 4f XPS results (Fig. 2b and c). This observation, which is consistent with prior studies of Ta_3_N_5_, once again highlights the challenges in obtaining O-free but N-rich metal nitrides.

**Fig. 3:**
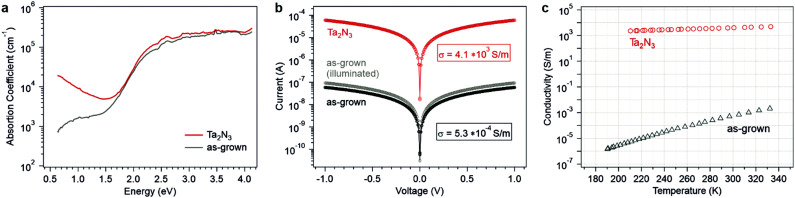
(a) Absorption coefficients of as-grown Ta_2_N_3_(O) and Ta_2_N_3_ measured by photothermal deflection spectroscopy. (b) Current–voltage characteristics (open circles) that were used to extract the in-plane electrical conductivities. (c) Temperature dependence of electrical conductivity on as-grown Ta_2_N_3_(O) and Ta_2_N_3_ films.

The optical absorption of as-grown Ta_2_N_3_(O) and Ta_2_N_3_ films were first analyzed by UV-vis spectroscopy. In order to eliminate thin film optical interference, both the transmission and reflectivity profiles were measured at a 15° angle of incidence, then the film absorbance was extracted (Fig. S11, ESI†). For both types of films, a strong absorption onset was observed at ∼650 nm (1.9 eV), though the Ta_2_N_3_ film exhibited a slightly sharper absorption edge following the reactive NH_3_ annealing treatment compared to the as-grown Ta_2_N_3_(O) sample. To achieve a higher dynamic range in analyzing the absorption coefficient, the background-free photothermal deflection spectroscopy (PDS) technique was used to probe the sub-bandgap region (Fig. 3a). The stoichiometric Ta_2_N_3_ film exhibits a Drude-like absorption feature that increases in strength towards lower photon energies. This phenomenon is consistent with free carrier absorption, and suggests a very high carrier concentration within the bixbyite Ta_2_N_3_. Interestingly, such a characteristic absorption feature was not observed for the as-grown Ta_2_N_3_(O) film that contained 11.6% lattice oxygen. Instead, a weaker sub-gap optical absorption that increased and plateaued with increasing photon energy was observed. Such an absorption feature, which is here found in the range of 0.6–1.5 eV, is consistent with optical transitions to or from electronic states within the bandgap. Hence, oxygen incorporation in Ta_2_N_3_ appears to significantly diminish the free carrier concentration within the material and the optical spectra of Ta_2_N_3_ and Ta_2_N_3_(O) resemble those of a degenerate and a non-degenerate semiconductor, respectively.

To further understand the role of oxygen on the electronic properties of Ta_2_N_3_, in-plane electrical conductivity measurements were performed. For this purpose, interdigitated Ti/Au contacts (20/80 nm) were evaporated atop as-grown Ta_2_N_3_(O) (Fig. S12, ESI†). For Ta_2_N_3_ films after reactive annealing in NH_3_, a significantly increased conductivity was discernible by a handheld multimeter. Therefore, two Ti/Ta contacts (10/50 nm) separated by 5 mm were prepared. As shown in Fig. 3b, ohmic behavior was observed for both samples, albeit with a drastic difference in conductivity, *σ*, of 5.3 × 10^−4^ and 4.1 × 10^3^ S m^−1^ for the Ta_2_N_3_(O) and Ta_2_N_3_ films, respectively. In other words, reduction in oxygen content within the films caused an electrical conductivity enhancement by seven orders of magnitude. Although the polycrystalline nature of the thin films investigated here precluded reproducible and statistically meaningful quantification of carrier concentration, type, and mobility by Hall effect measurements, Ta_2_N_3_ films exhibited negative Seebeck coefficients, thus confirming their n-type character. Furthermore, light illumination gave rise to an additional photoconductivity for the as-grown Ta_2_N_3_(O) sample, but this phenomenon was absent from Ta_2_N_3_. This result is consistent with the Drude-like feature observed in optical absorption measurements and indicates that the background carrier concentration in Ta_2_N_3_ was much higher than the photoexcited carrier concentration.

The temperature-dependent conductivities of Ta_2_N_3_(O) and Ta_2_N_3_ are shown in Fig. 3c. For the case of Ta_2_N_3_(O), a strong temperature dependence with *σ* increasing from 1.5 × 10^−6^ S m^−1^ at 190 K to 2.1 × 10^−3^ S m^−1^ at 332 K was observed (Fig. 3c). In contrast, only a very weak temperature dependence (approximately a factor of two change) was observed for Ta_2_N_3_ over the same temperature range. Taken together, optical absorption, photoconductivity, and temperature-dependent transport data provide strong evidence that Ta_2_N_3_(O) behaves as a non-degenerate semiconductor, while Ta_2_N_3_ exhibits the characteristics of a metallic conductor. Thus, it appears that oxygen incorporation plays a major role in reducing the carrier concentration within the material and may be used to widely tune its electronic and transport properties across the metal-semiconductor transition.

**Fig. 4:**
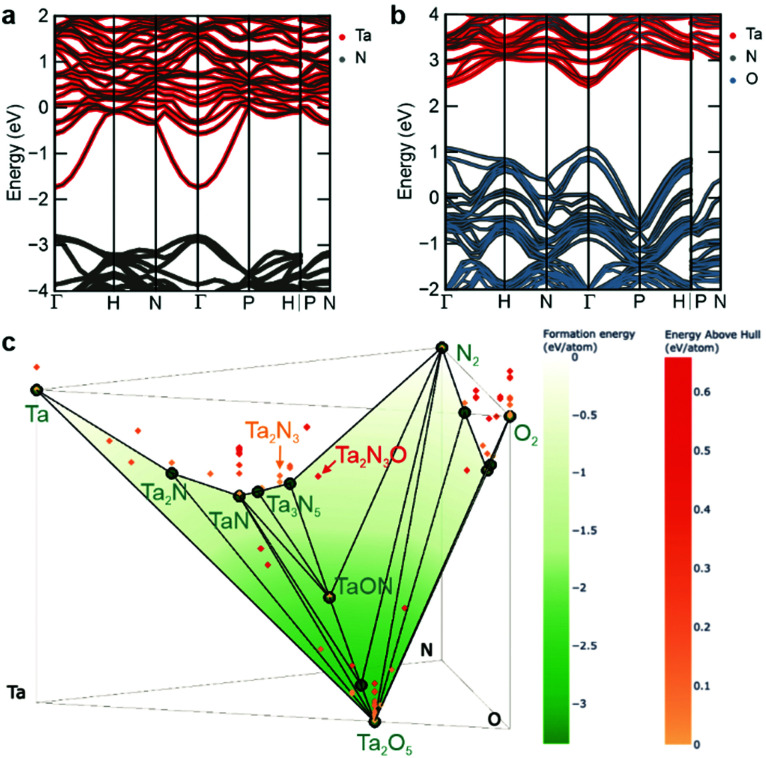
Predicted band structures for (a) bixbyite-type Ta_2_N_3_ and (b) Ta_2_N_3_O, where the oxygen atoms sit in interstitial positions, calculated by density functional theory using HSE06 hybrid functional. (c) Predicted convex hull phase diagram at 0 K for the Ta–N–O chemical system incorporating data from the Materials Project and supplemented with compatible data for the bixbyite-like Ta_2_N_3_ and Ta_2_N_3_O for the present work.

To rationalize these findings regarding the intrinsic metallic nature of Ta_2_N_3_ and the role of oxygen as a compensating impurity dopant, the electronic structure of Ta_2_N_3_, as well as an idealized model Ta_2_N_3_O were calculated by density functional theory (DFT) using HSE06 hybrid functionals.^41^ The electronic band structures of these two compounds are generated with *sumo*^42^ and *pymatgen*^43^ and shown in Fig. 4a and b, with the zero of the energy scale set to the Fermi level. Both compounds exhibit a series of high-lying Ta 5d bands that are separated from N 2p bands by an approximately 1.1 eV energy gap. For the bixbyite-type Ta_2_N_3_, the Fermi level is located within the Ta 5d bands, which can be attributed to the average donation of half a valence electron per Ta atom due to its +4.5 average oxidation state within Ta_2_N_3_. This result is in excellent agreement with the experimental observations of the metallic character of Ta_2_N_3_. Indeed, considering the +4.5 average oxidation state of Ta, the n-type doping level in Ta_2_N_3_ should be on the order of 10^22^ cm^−3^ without accounting for electronically active structural defects or impurity doping, making it a degenerately-doped semiconductor.

Comparative evaluation of the predicted band structure for the model stoichiometric Ta_2_N_3_O compound, with all oxygen sitting at the ordered vacancy sites of the bixbyite structure reveals several important similarities and differences. Like Ta_2_N_3_, the Ta_2_N_3_O system is characterized by an 1.5 eV internal electronic bandgap. As expected from the relative energetic positions of N 2p and O 2p orbitals, the valence band maximum retains a dominant contribution from N 2p, while O 2p states are introduced deeper within the valence band. Although metallic character is also predicted for Ta_2_N_3_O, the Fermi level shifts downwards from the Ta-dominant conduction band into the lower energy N/O dominant valence band of the material, indicating p-type metallic conduction. However, it should be noted that since this calculation is performed for the idealized oxynitride system possessing full oxygen occupation of all ordered vacancy sites of the bixbyite structure, it represents the maximum electronic structure change and Fermi level shift that can be expected for oxygen incorporation into Ta_2_N_3_. At intermediate oxygen content, the Fermi level is predicted to lie within the bandgap. Interestingly, this provides the prospect not only for oxygen to drive the system across the metal-to-semiconductor transition, but also to modulate the conductivity from n-type to p-type as the oxygen content increases. In contrast to conventional doping, such strong conductivity changes are expected to occur at very high oxygen concentrations, where there is a concomitant change of the electronic structure of the material itself. In the ternary ZnSnN_2_ system that adopts the wurtzite structure, Pan *et al.* suggested that oxygen impurity could pair with excess Zn defects and thus lower the net doping concentration from 10^20^ to 10^17^ cm^−3^ range in highly off-stoichiometric material.^44^ Kim *et al.* have also reported the similar compensation effect of lattice oxygen on Mg-rich MgZrN_2_ with NaCl-like structure.^45^ These results, together with the drastic modification to the Ta_2_N_3_ electronic structure by interstitial oxygen incorporation presented in this work, highlight the potential in designing functional transition metal nitrides with desired electronic properties *via* controlling the oxygen impurity content, as well as configurational disorder related to both cations and anions.

Despite the intriguing electronic structure computed for the idealized Ta_2_N_3_O oxynitride phase, no experimental evidence for its formation or for metallic conduction with increasing oxygen content were observed under any processing conditions. Indeed, as described above, introduction of additional oxygen into the process gas eventually resulted in decreasing structural quality of films and the ultimate formation of amorphous material. To better understand this synthetic inaccessibility of stoichiometric and crystalline Ta_2_N_3_O, DFT was used to compute the energy landscape for the ternary Ta–N–O system at 0 K. Fig. 4c shows a phase diagram representing the formation energy as a function of composition (incorporating data from the Materials Project^46^), with thermodynamically stable compounds indicated as solid green points. Higher energy phases, which may be either metastable or unstable (see below), are indicated as red shaded points lying above the hull. Interestingly, both Ta_2_N_3_ and Ta_2_N_3_O are predicted to be thermodynamically unstable, with above-hull energies of 0.11 and 0.61 eV per atom, respectively.

An important feature of nitride compounds is their exceptionally large energetic window of metastability, which originates from the high cohesive energy of metal–nitrogen bonds.^8^ By comparison, oxides exhibit a much smaller energetic range over which metastable phases can be realized. To quantify this, the so-called amorphous limit was recently established.^47^ In particular, by comparing the free energies of crystalline phases to the corresponding amorphous state it is possible to quantify the energetic windows of synthetically accessible crystalline polymorphs of metastable compounds. In this context, the amorphous limit for TaN is comparatively large, with a computed value of 0.46 eV per atom, while the limit for Ta_2_O_5_ is 0.2 eV per atom. Using these representative values, we conclude that the above-hull energy of Ta_2_N_3_ of 0.11 eV per atom falls well within the energetic range of synthetically accessible compounds, while the corresponding value of 0.61 eV per atom for Ta_2_N_3_O significantly exceeds the expected amorphous limit. This prediction is in excellent agreement with our experimental findings, in which crystalline Ta_2_N_3_ and Ta_2_N_3_(O) compounds can be synthesized using the non-equilibrium reactive sputtering approach. However, with increasing oxygen introduced to the system, amorphous films, rather than crystalline Ta_2_N_3_O are observed (Fig. 1b).

**Fig. 5:**
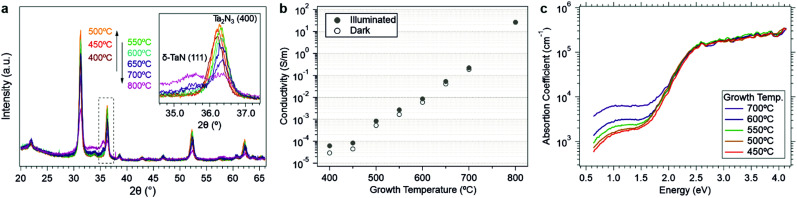
(a) XRD patterns of as-grown Ta_2_N_3_(O) films deposited at different substrate temperatures. Inset: Enlarged region of Ta_2_N_3_(400) diffraction peaks and the impurity peak associated with δ-TaN(111) diffraction. (b) Measured in-plane electrical conductivities of as-grown Ta_2_N_3_(O) films with different growth temperatures. (c) Absorption coefficients of as-grown Ta_2_N_3_(O) films, measured by photothermal deflection spectroscopy, as a function of growth temperature.

To further explore the metastability of these compounds, the influence of growth temperature on film structure was investigated. In general, as temperature increases, entropic contributions to the free energy will increasingly favor the amorphous state of matter. Thus, the 0 K calculations of free energies of amorphous *versus* crystalline polymorphs provide a good indication of the amorphous limit and the basic feasibility of a metastable material to be synthesized. However, at increasingly high temperatures, metastable phases can become kinetically unlocked and films will be driven towards lower energy states, either *via* phase segregation or *via* amorphization. Fig. 5a shows the experimental XRD patterns collected as a function of growth temperature between 400 and 800 °C. In contrast to the conventional structure zone model, XRD measurements revealed that the film crystallinity reached a maximum at 500 °C, above which an increasingly intense amorphous background between 2*θ* = 32–35° was observed. This change was accompanied by the appearance of a new diffraction peak at 35.5°, which can be assigned to the (111) reflection of δ-TaN. Consistent with this assignment, XPS analysis of the sample gown at 800 °C was characterized by an additional lower binding energy doublet at 23.2 and 25.1 eV in the Ta 4f region, which indicates the presence of the lower Ta^3+^ oxidation state of δ-TaN (Fig. S14, ESI†).

Along with the emergence of the δ-TaN and amorphous phases at growth temperatures >500 °C, a monotonic shift of the remaining bixbyite Ta_2_N_3_ diffraction peaks toward larger angles was also identified by XRD (Fig. 5a). Since oxygen incorporation into the bixbyite structure leads to unit cell expansion, the smaller lattice constant at increasing growth temperature suggests reduced oxygen content within the remnant crystalline Ta_2_N_3_(O). Such a finding would be consistent with local compositional inhomogeneities, which result in stronger driving forces for amorphization in oxygen-rich regions and retention of Ta_2_N_3_ in oxygen deficient regions. While verification of this hypothesis will require future (spectro-)microscopic investigations of the material, our observation is that high growth temperature drives the system towards a phase segregated amorphous state with increasingly large concentrations of the thermodynamically stable δ-TaN phase. This is in excellent agreement with theoretical calculations of the metastability of bixbyite Ta_2_N_3_ and Ta_2_N_3_O, as well as the prior report of Ta_2_N_3_ decomposition into δ-TaN above 750 °C.^19^

Investigations of the optoelectronic properties of Ta_2_N_3_(O) films as a function of growth temperature provide additional support for the conclusions derived from the structural study described above, as well as opportunities for broadly tailoring film properties. As shown in Fig. 5b (and in Fig. S13, ESI†), the conductivity *σ* increased from 2.92 × 10^−5^ to 26.2 S m^−1^ – a six order of magnitude variation – when the growth temperature was raised from 400 to 800 °C. Corresponding PDS data reveal that the sub-bandgap absorption strength increased with the growth temperature (Fig. 5c). These trends with growth temperature are consistent with the structural results described above and may be described by two parallel factors: (i) the increasing fraction of metallic δ-TaN at temperatures above 500 °C, as detected by the emergence of the (111) δ-TaN diffraction peak, and (ii) the reduced oxygen content in the crystalline Ta_2_N_3_(O) phase fraction with increasing temperature, as detected by the shifting of Ta_2_N_3_(400) peak to larger angles.

These two parallel effects of oxygen incorporation and phase segregation can explain prior discrepancies in the reported optoelectronic properties of bixbyite Ta_2_N_3_, which include differing assignments of the material as a semiconductor or a metal. In the first account by Coyne *et al.* in 1968,^27^ an optical bandgap varying between 1.95 and 2.57 eV and a 0.1–0.3 eV energy barrier for electrical conduction were described, leading to the conclusion that the material is a semiconductor. In contrast, Ganin *et al.* reported their Ta_2_N_3_ thin films to be black in color and metallically conducting.^17^ Our results confirm that bixbyite Ta_2_N_3_ is a metastable compound exhibiting strong optical absorption above an ∼1.9 eV onset, but is intrinsically a metallic conductor with its Fermi level lying within the conduction band. Our observation that oxygen impurities can induce a metal-to-semiconductor transition can account for the conflicting assignment of the electronic nature of the material. Furthermore, we note that the existence of a window of transparency for our NH_3_-annealed Ta_2_N_3_ film in the near infrared range is in accordance with the degenerate nature of the material and may provide opportunities for its use as a semi-transparent contact layer. Likewise, the ability to extend this transparency window while controllably reducing its electrical conductivity suggests potential applications for the creation of tailored photonic systems. Moreover, the ∼1.9 eV optical absorption onset and highly tunable conductivity of Ta_2_N_3_ offer intriguing prospects for solar energy conversion applications. Preliminary tests using as-grown semiconducting Ta_2_N_3_(O) as a photoelectrode revealed that the material is photoactive (Fig. S15, ESI†). An anodic photocurrent onset was observed at ∼0.8 V *vs.* RHE in the presence of 0.1 M K_4_Fe(CN)_6_ as a sacrificial hole acceptor, consistent with the n-type character of the film. While the ∼5 μA cm^−2^ photocurrent density is much lower than possible for a material with such an absorption characteristic, we note that this result was achieved with no specific optimization for photoelectrochemical function. In particular, the films are only ∼90 nm thick and not optically dense, both the back contact and surface native oxide may introduce significant out of plane transport barriers, and the oxygen concentration has not been optimized for maximizing photocurrent. Nevertheless, the observed anodic photoactivity confirms the n-type semiconducting character of the as-grown material and provides a basis for future investigations of photoelectrochemical function.

In 2016, Salamon *et al.* found that the N/Ta ratio of sputtered films increases with the nitrogen partial pressure in the process gas,^19^ and the N-rich films crystallize to Ta_2_N_3_ but decompose into δ-TaN at >750 °C. Not only did they find that the crystallinity of Ta_2_N_3_ films improves upon vacuum annealing, but also that the electrical conductivity increases from 2.9 S m^−1^ for the as-grown film to 2.2 × 10^4^ S m^−1^ for the sample annealed at 750 °C.^28^ Such phenomena were attributed to the out-diffusion of excess nitrogen present at the grain boundaries of polycrystalline films. Since this excess nitrogen was assumed to introduce insulating barriers between otherwise metallic Ta_2_N_3_ nanocrystals, the effect of vacuum annealing was to increase the macroscopic film conductivity by reducing inter-grain transport barriers. However, in the present work, the lack of free carrier absorption in the as-grown films (Fig. 3a), as well as their photoconductive response (Fig. 3b), excludes the presence of metallic grains separated by insulating grain boundaries. Rather, the correlation between decreasing oxygen content and decreasing lattice constant provides strong evidence that the oxygen is incorporated within the Ta_2_N_3_ lattice of as-grown material and that its concentration decreases with increasing NH_3_ annealing temperature. Taken together, the systematic decrease of lattice constant, increase of sub-bandgap optical absorption, and increase of electrical conductivity after reactive annealing in NH_3_ are consistent with the proposed impact of interstitial oxygen in decreasing the free carrier concentration in Ta_2_N_3_. This conclusion is also supported by first principles calculations by including oxygen in the structural vacancy sites. The percolation model proposed by Salamon *et al.* may be relevant to films possessing nitrogen-rich grain boundaries, but does not describe the oxygen-induced conductivity changes observed here. Finally, to our best knowledge, the only other report of bixbyite Ta_2_N_3_ is by Dekkers *et al.*, who used TaCl_5_ and NH_3_ as atomic layer deposition precursors.^48^ In that work, a conductivity of ∼1.3 × 10^4^ S m^−1^ was determined for Ta_2_N_3_ crystallized at 500 °C. The high conductivity values found in these prior studies are in agreement with low oxygen content films, similar to those achieved here through post-synthetic reactive NH_3_ annealing. In addition, the observation of increasing conductivity with increasing post-deposition vacuum annealing temperature suggests an alternative strategy for driving oxygen out of the material to control its conductivity. However, special attention needs to be placed on the competitive phase decomposition to δ-TaN, which is a feature of the metastability of this compound but, as presented above, can be suppressed *via* annealing in NH_3_.

## Conclusions

In this work, we prepared homogeneous and compact tantalum sesquinitride (Ta_2_N_3_) thin films by reactive sputter deposition and characterized their structural, optical, and electronic transport characteristics as a function of growth conditions, as well as after post-synthetic reactive annealing in NH_3_. Comparison of computed phase stabilities with structural characteristics of these films provided significant insight into the metastable nature of Ta_2_N_3_, as well as the role of incorporated oxygen on the energetic window of metastability. Here, we found that a controlled dose of oxygen (∼0.65%) in the process gas mixture was imperative to the successful formation of the bixbyite phase, which can be explained by the oxygen inductive effect stabilizing the metastable Ta_2_N_3_. While stoichiometric Ta_2_N_3_ was theoretically predicted to be a degenerate semiconductor, as-synthesized films were found to exhibit semiconducting character with an optical absorption onset of ∼1.9 eV and notable photocurrent generation. The origin of this apparent discrepancy is the incorporation of ∼11.6 at% oxygen into the films, which induced a metal-to-semiconductor transition. However, post-deposition thermal annealing in NH_3_ yielded a significantly reduced oxygen content, which was accompanied by a seven order of magnitude increase in the electrical conductivity. As a consequence, controlled oxygen incorporation in this rarely studied compound provides significant opportunity to broadly tailor electronic transport characteristics of relevance for applications ranging from photochemical energy conversion to advanced photonics.

Beyond controlling oxygen content to tailoring optoelectronic properties, the possibility of synthesizing an entirely new oxynitride composition, Ta_2_N_3_O, was explored. However, theoretical calculations predicted the crystalline phase of this material, which would be achieved by ordered oxygen incorporation into structural vacancies of the bixbyite structure, to be synthetically inaccessible due to its large formation energy. Experimental observations confirm this prediction, with increasing oxygen content leading to the formation of amorphous films. Likewise, increasing the growth temperature favors segregation of films into thermodynamically preferred δ-TaN and an amorphous tantalum oxynitride, which is consistent with the predicted metastability of the pure nitride Ta_2_N_3_. Overall, the excellent agreement between theory and experiment regarding synthetic accessibility of metastable compounds within the Ta–N and the Ta–N–O composition spaces highlights the powerful potential of modern computational models for rationally guiding synthetic strategies and materials discovery approaches.

## Experimental section

### Deposition of Ta_2_N_3_ thin films

Prior to each deposition, double side polished amorphous SiO_2_ substrates (Siegert Wafer GmbH) were cleaned consecutively in 1 vol% Hellmanex, acetone, and isopropanol using an ultrasonic bath. For elemental analysis of oxygen content, Si(100) wafers were used instead of fused silica substrates. The n-type doped Si wafers were cut to small pieces and cleaned by dry N_2_ flow. The substrate holder was rotated at 10 rpm, and was heated by an infrared lamp to 500 °C at a ramp rate of 10 °C min^−1^. The base pressure in the process chamber (PVD 75, Kurt J. Lesker) remained below 8 × 10^−8^ torr and 3 × 10^−7^ torr at room temperature and 500 °C, respectively.

A 2-inch diameter Ta target (Kurt J. Lesker, 99.95%) placed 20 cm away from the substrate was initially sputtered by Ar plasma for 15 minutes. During this cleaning period, the process gas contained Ar (9.5 mTorr, Linde Electronics GmbH, 99.9999%) and a 60 W DC sputtering power was applied to the Ta target. This was followed by 10 minutes of target conditioning period, during which the 6.7 mTorr process gas consisted of argon (2.2 mTorr), nitrogen (4.4 mTorr, Linde Electronics GmbH, 99.9999%), and oxygen (∼0.04 mTorr, Linde Electronics GmbH, 99.9999%). The sputtering power was switched to pulsed DC mode at 100 kHz repetition rate and 98% duty cycle, with a 50 W average power, which equilibrated at a cathodic potential of 560 V and a sputtering current of 0.09 A. The actual deposition period was started by opening the substrate shutter only when the target potential had reached a steady state. To investigate the influence of O_2_ on the as-grown tantalum nitride, a series of thin films was grown on amorphous SiO_2_ at 500 °C substrate temperature with the oxygen flow rate varying between 0–0.6 sccm, which was equivalent to 0–1.93% O_2_ concentration in the process gas.

NH_3_ annealing was performed in a quartz tube with a horizontal tube furnace. The samples were heated at a 60 °C min^−1^ ramp rate under 100 sccm constant flow of NH_3_ (Linde Electronics GmbH, 99.999%) for 3 h. After the annealing procedure, the samples were allowed to cool down naturally in NH_3_ flow until the temperature was below 150 °C.

### Material characterization

Structural characterization was performed by an X-ray diffractometer (SmartLab, Rigaku) using Cu K_α_ radiation at 1° grazing incident angle. The 2*θ* diffraction angle was scanned between 15–70° with 0.02° step size. X-ray reflectivity (XRR) measurements were done with the same instrument, with the 2*θ*/*ω* angle scanned between 0–4° with 0.01° step size. For analysis of XRR data, we note that the much larger atomic mass of Ta compared to O and N means that film density is not significantly affected by the O content (*i.e.* the predicted density of Ta_2_N_3_O would be 11.34 g cm^−3^ compared to 11.33 g cm^−3^ for Ta_2_N_3_). Film morphology was inspected using a scanning electron microscope (NVision 40, Zeiss). X-ray photoemission spectroscopy (XPS) data were acquired using a SPECS Phoibos 100 spectrometer equipped with an Al K_α_ source (*hν* = 1486.69 eV) and a hemispherical electron energy analyzer. XPS binding energies were calibrated using adventitious alkyl carbon signals by shifting the C 1s peak to 284.8 eV.

Optical transmission and specular reflection spectra were collected with an UV-vis spectrometer (Lambda 900, PerkinElmer) at 15° incident angle. Photothermal deflection spectroscopy was performed with a home-built system, in which the sample was immersed in perfluorohexane and illuminated at a 90° incident angle by a 9 Hz monochromatic xenon/halogen light source. The probe beam was provided by a 635 nm laser diode (CPS635, Thorlabs) and propagated parallel to and near the sample surface. The deflection of the probe beam, as a function of incident wavelengths, was detected by a quadrant photodiode connected to a lock-in amplifier.

Rutherford back-scattering (RBS) analysis of Ta_2_N_3_(O) and Ta_2_N_3_ on silicon substrates was conducted using 2 MeV ^4^He^+^ ion at normal incidence with a 170° total scattering angle. Data simulation was done with SIMNRA software. A small amount (<1%) of Ar was detected together with some extent of Ta interdiffusion into silicon. More precise quantification of N and O was performed by elastic recoil detection (ERD) analysis of the same sample using 36 MeV I^+^ ion probe. Both the incident and detection angles were 67° with respect to surface normal. The depth scale does not make any assumptions regarding the density of the material, but dividing by the density in atoms cm^−3^ will provide the depth in units of cm. It should be noted that RBS detected trace sulfur and iron following NH_3_ annealing treatment, which are not included as predominant constituents in the elemental composition calculation in Table S1 (ESI†). The oxygen content in the sample was reduced in the ERD result, while the Ta content remained unchanged. A slight increase in N content was observed in the bulk of the film.

### Electrical conductivity measurement

Interdigitated contacts were first patterned on as-grown tantalum nitride films by photolithography. For this, a layer of inversion image photoresist (AZ 5214E, MicroChemicals) was spin-coated onto the clean sample surface. After light exposure through a patterned photomask and subsequent development, e-beam evaporation was used to deposit consecutively 20 nm Ti and 80 nm Au. The remaining photoresist was then removed by acetone. Each contact consisted of thirteen 3.86 mm-long strips, with a minimum distance between the contacts of 60 μm.

The in-plane electrical conductivity can be calculated *via* the slope of the current–voltage line with the following formula:
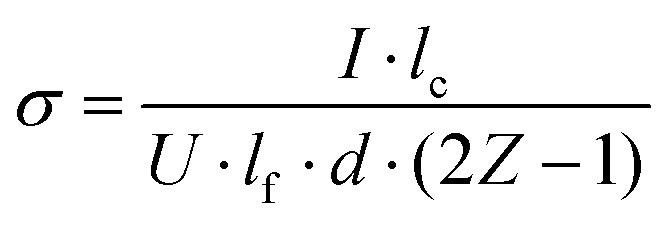
in which *U* is the applied voltage across the two contacts, *I* is the measured current, *l*_c_ is the distance between contacts, *l*_f_ is the length of each contact strip, *d* is the thickness of the thin film sample, and *Z* is the number of strip pairs.

The photoconductivity was measured using a halogen lamp (MLC-150C, Motic) with a 3500 K color temperature, with a 15 min settling time before each measurement. The temperature dependence of the electrical conductivity was examined between 190–330 K, from low to high set temperature points, in a home-built setup.

### Photoelectrochemical measurement

The test was conducted using an AM 1.5G solar simulator (HAL-320, Asahi Spectra) in a three-electrode configuration. A Ta_2_N_3_(O) thin film on a degenerately doped Si(100) substrate and a Pt wire served as the working and counter electrode, respectively, while a Ag/AgCl electrode immersed in 3 M KCl solution was used as the reference electrode. The pH 9.35 buffer solution consisted of 0.1 M H_3_BO_3_ and 0.05 M NaOH, and the addition of 0.1 M K_4_Fe(CN)_6_ served as a sacrificial hole acceptor. Current–potential characteristics were recorded with a potentiostat (SP-150, Bio-Logic).

### First principles calculations

Calculations were performed with VASP 5.4.4^49^ and the plane-augmented wave method and pseudopotentials matching the Materials Project^46^ standard set with plane-wave cut-off of 520 eV and *k*-point reciprocal density of 64 *k*-points per inverse Å^3^ relaxations and 100 *k*-point reciprocal per Å^3^ for total energy calculations. To fully reproduce these calculations, please consult the workflows defined in the *atomate*^50^ and *pymatgen*^43^ code, specifically the “MPRelaxSet”, “MPStaticSet”, and “MPHSEBSSet” classes.

For the electronic structure calculations, a crystal structure of bixbyite Ta_2_N_3_ and the same structure with interstitial oxygen atoms added for the Ta_2_N_3_O phase, were first relaxed with the PBEsol energy functional,^36^ and then the electronic structure was calculated using the HSE06 functional^41^ known to predict reasonably accurate band gaps in semiconductors to within a few tenths of eV.

For the phase diagram, calculations, similar geometry optimizations and total energy calculations were performed, except using the PBE energy functional^51^ to retain compatibility with existing Materials Project data. Alternative functionals were also explored for the Ta–N phase including the SCAN energy functional with differences in total energy of the order of 2 meV per atom, suggesting the PBE is sufficient for characterization of phase stability in this system.

## Conflicts of interest

There are no conflicts to declare.

## Supplementary Material

MH-008-D1MH00017A-s001
