# Supplementary material for: Metastable Ta2N3 with highly tunable electrical conductivity via oxygen incorporation
Source: Mater Horiz. 2021 Apr 1;8(6):1744–55. doi: 10.1039/d1mh00017a (PMC8186396; doi:10.1039/d1mh00017a)
Supplement: MH-008-D1MH00017A-s001 [file MH-008-D1MH00017A-s001.pdf]

Electronic Supplementary Material (ESI) for Materials Horizons.
This journal is © The Royal Society of Chemistry 2021

Supplementary Information

Metastable Ta_2N_3 with Highly Tunable Electrical Conductivity via Oxygen Incorporation

Chang-Ming Jiang,[a] Laura I. Wagner,[a] Matthew K. Horton,[bc] Johanna Eichhorn,[a] Tim Rieth,[a] Viktoria F. Kunzelmann,[a] Max Kraut,[a] Yanbo Li,[d] Kristin Persson,[bc] Ian D. Sharp*[a]

[a] Walter Schottky Institute and Physics Department, Technische Universität München, 85748 Garching, Germany
[b] Energy Technologies Area, Lawrence Berkeley National Laboratory, Berkeley, CA 94720, USA
[c] Department of Materials Science and Engineering, University of California, Berkeley, Berkeley, CA 94720, USA
[d] Institute of Fundamental and Frontier Sciences, University of Electronic Science and Technology of China, Chengdu 610054, PR China

*Corresponding author: sharp@wsi.tum.de

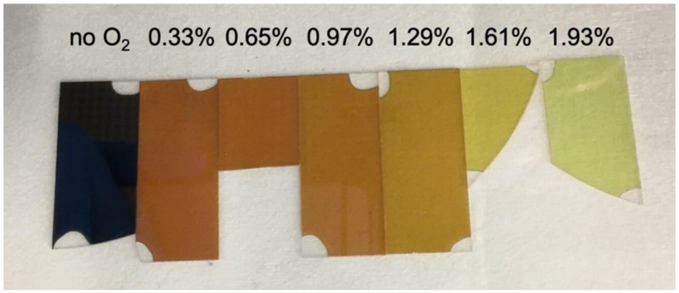

Fig. S1 Optical photograph of as-grown tantalum nitride thin films on amorphous SiO_2 substrates. Without intentionally added O_2 in the process gas the film appearance was metallic. Brown-orange films were obtained when < 1% O_2 was added in the process gas during the reactive sputtering process.

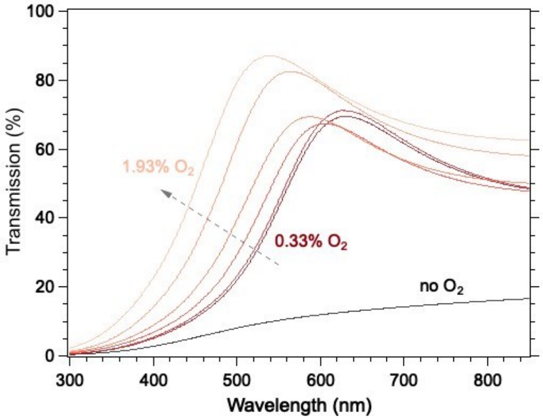

Fig. S2 UV-Vis transmission spectra (15° angle of incidence) of as-grown tantalum nitride films. A monotonic shift of absorption onset toward shorter wavelengths was observed as the O_2 concentration in the process gas increased.

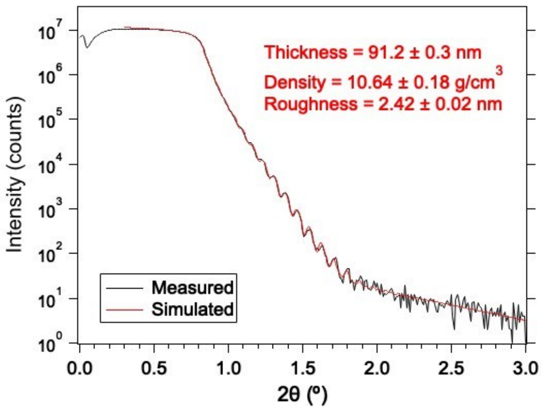

Fig. S3 X-ray reflectivity (XRR) measurement result of an as-grown Ta_2N_3 film on silicon substrate. The simulation was performed between 0.3 – 3.0° using the Rigaku SmartLab Studio II software.

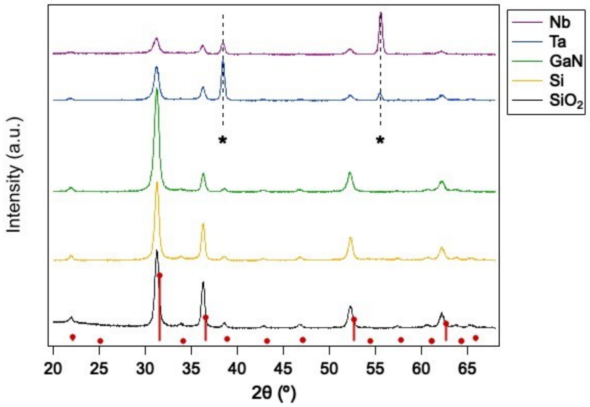

Fig. S4 Grazing-incident XRD patterns of as-grown Ta_2N_3 thin films on different substrates. Red sticks: Bragg-reflection positions and intensities of bixbyite-type Ta_2N_3. The diffraction background from Nb and Ta substrates are indicated with asterisks.

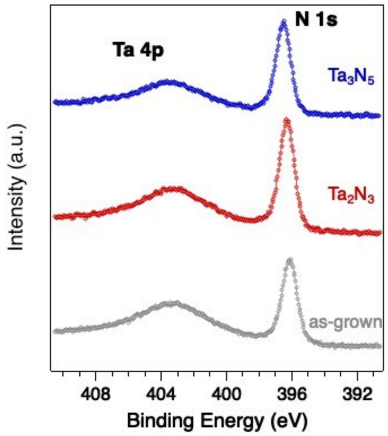

Fig. S5 N 1s and Ta 4p XPS spectra of as-grown $Ta_2N_3(O)$ and after NH_3 annealing treatments.

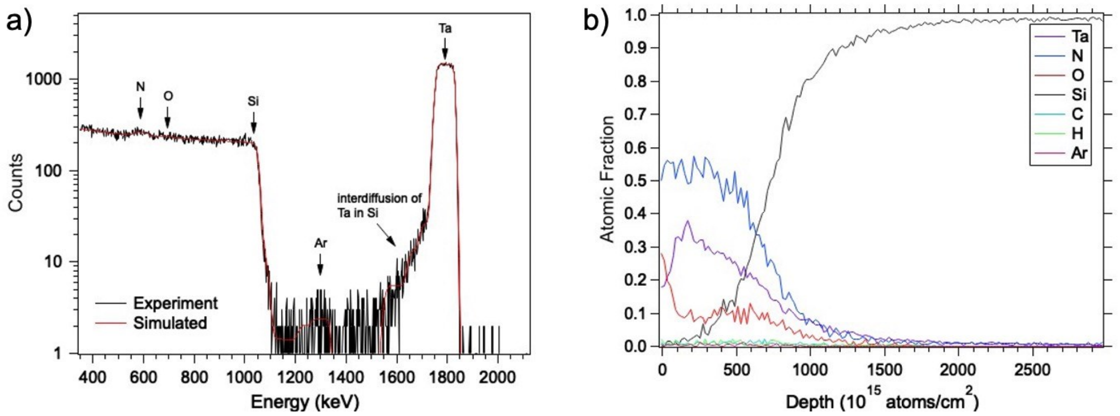

Fig. S6 (a) RBS and (b) ERD results acquired from an as-grown $Ta_2N_3(O)$ on silicon substrate.

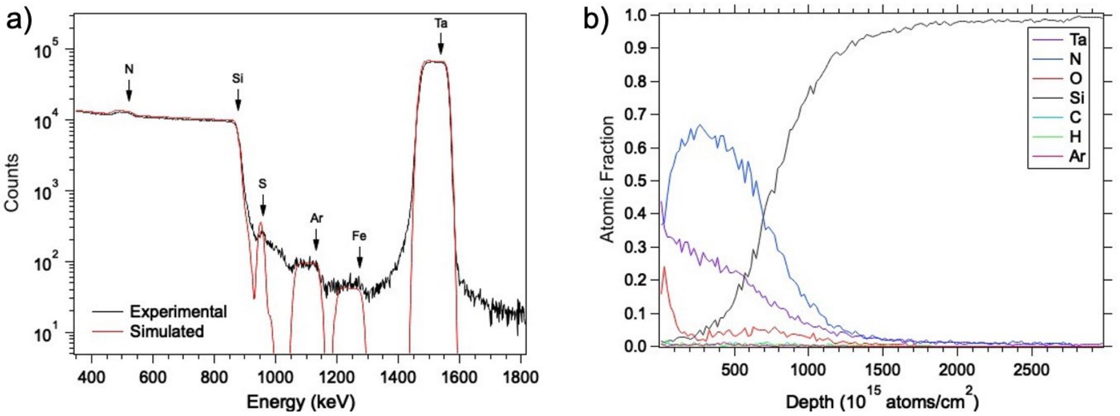

Fig. S7 (a) RBS and (b) ERD results acquired from Ta_2N_3 after NH_3 annealing treatment.

Table S1 The average bulk elemental composition of Ta_2N_3 films.

**Sample**	**Atomic %**					
	**Ta**	**N**	**O**	**H**	**C**	**Ar**
as-grown *$Ta_2N_3(O)$*	*33.3*	*51.7*	*11.6*	*1.5*	*1.5*	*0.4*
NH_3 annealing @ 750°C	*28.5*	*63.5*	*5.8*	*0.9*	*0.8*	*0.5*

Table S2 Unit cell parameters of Ta_2N_3 and Ta_2N_3O.

Sample	Lattice constant (Å)	Atom	x	y	z	Density ($g \cdot cm^{-3}$)
Ta_2N_3 (reference)	9.8205	Ta1	1/4	1/4	1/4	11.327
		Ta2	-0.0280	0	1/4	
		N	0.3770	0.1530	0.3770	
Ta_2N_3 (calculated)	9.7338	Ta1	0	1/4	0.2893	11.632
		Ta2	0	0	0	
		N	0.0951	0.1468	0.6296	
Ta_2N_3O (calculated)	9.9453	Ta1	0	1/4	0.2643	11.338
		Ta2	0	0	0	
		N	0.1182	0.1291	0.6263	
		O	0.1263	0.1263	0.1263	

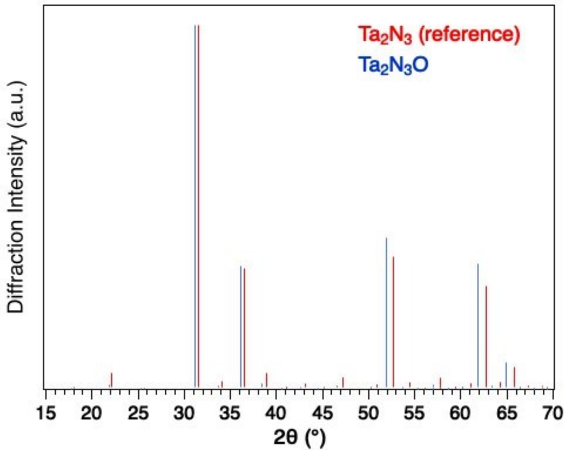

Fig. S8 X-ray diffractograms of Ta_2N_3 and Ta_2N_3O calculated based on the unit cell parameters in Table S2.

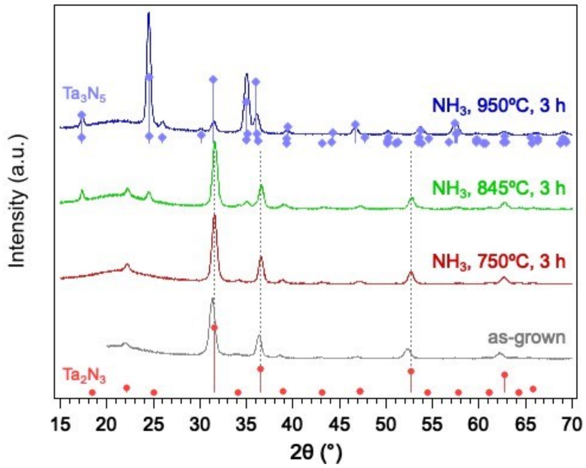

Fig. S9 XRD patterns of as-grown $Ta_2N_3(O)$ on SiO_2 substrate and after NH_3 annealing treatment at different temperatures.

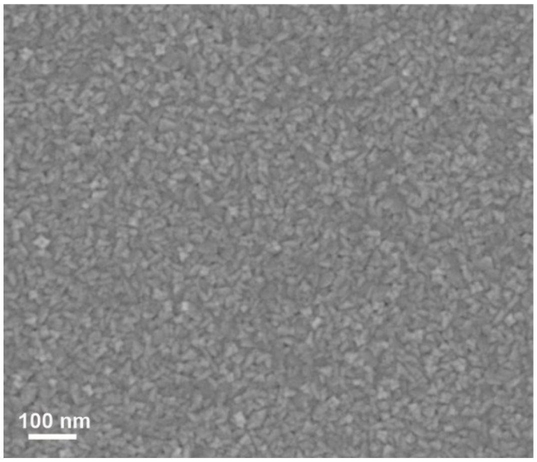

Fig. S10 SEM image of a Ta_2N_3 film on silicon after NH_3 annealing at 750 °C for 3 h.

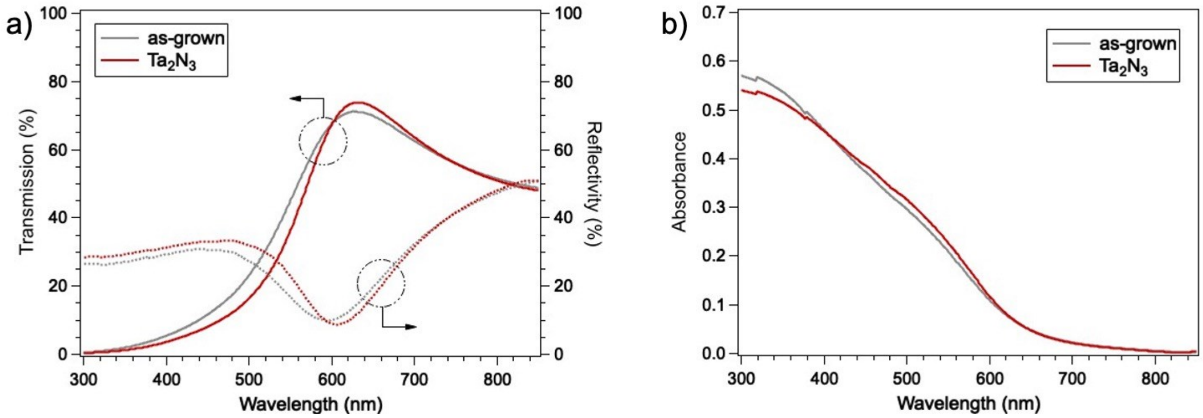

Fig. S11 (a) UV-Vis transmission and reflectivity spectra measured from as-grown $Ta_2N_3(O)$ and Ta_2N_3 thin films. (b) Film absorbances calculated based on the UV-Vis results.

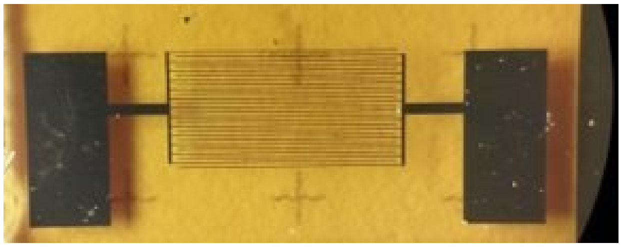

Fig. S12 Photograph of a pair of interdigitated contacts on $Ta_2N_3(O)$ thin film grown on SiO_2.

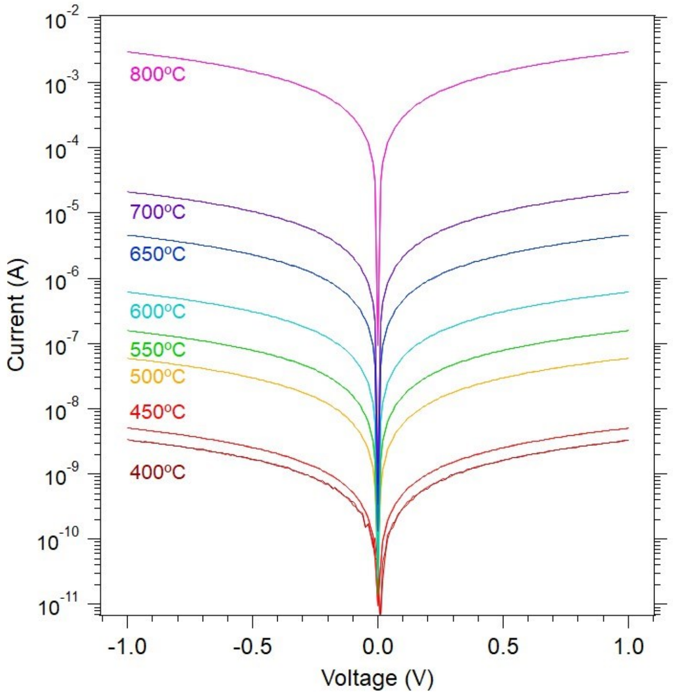

Fig. S13 Current-voltage characteristics of as-grown $Ta_2N_3(O)$ deposited at different temperatures.

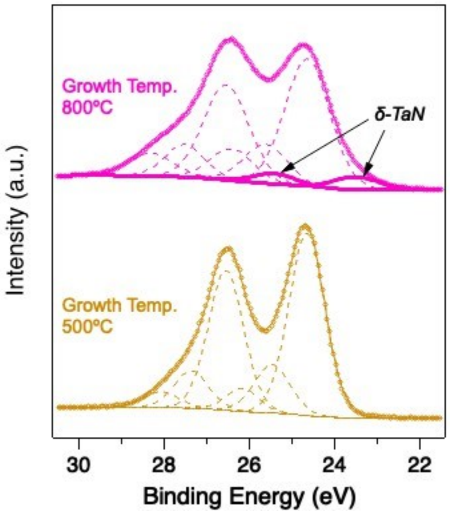

Fig. S14 Ta 4f region XPS spectra of as-grown $Ta_2N_3(O)$ films deposited at 500 °C and 800 °C substrate temperatures.

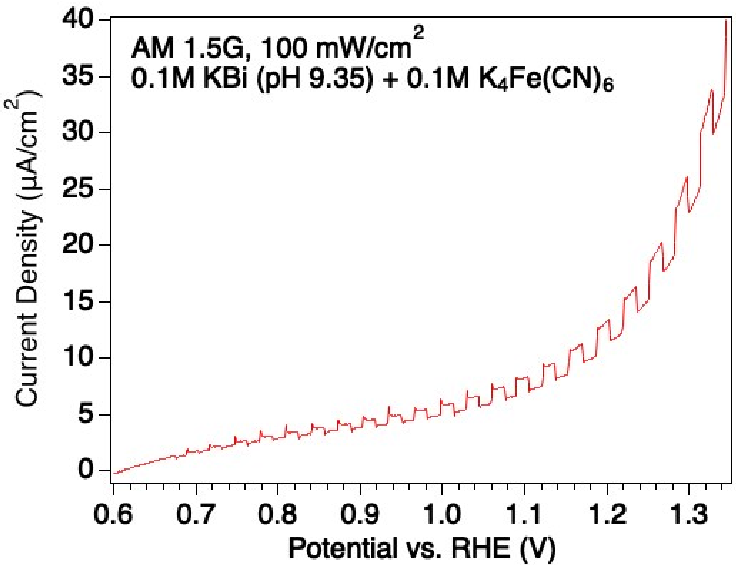

Fig. S15 Linear sweep voltammetry measurement result of a Ta_2N_3(O) photoelectrode on Si substrate under chopped illumination by simulated AM1.5G irradiation at 100 mW/cm^2 intensity.
